# Vertebral Artery Dissection and PICA-Territory Stroke Associated with Chronic Atlantoaxial Structural Abnormality: A Case Report

**DOI:** 10.3390/neurolint18090173

**Published:** 2026-09-14

**Authors:** Mahika Khurana, Seyed Mostafa Razavi, Balaganesh Natarajan, Eman Elmasry, Ahmed Abd Elazim

**Affiliations:** Sanford Health, Sioux Falls, SD 57104, USA; seyedmostafa.razavi@sanfordhealth.org (S.M.R.); balaganesh.natarajan@sanfordhealth.org (B.N.); elmasry.eman@yahoo.com (E.E.); ahmed.abdelazim@sanfordhealth.org (A.A.E.)

**Keywords:** vertebral artery dissection, posterior circulation stroke, PICA infarction, atlantoaxial abnormality, seronegative inflammatory arthritis, craniovertebral junction

## Abstract

Upper cervical structural disease can alter vertebral artery biomechanics, although a mechanical contribution cannot be established by anatomical proximity alone. We describe an older woman with seronegative inflammatory arthritis and chronic atlantoaxial abnormality who developed abrupt vertigo, nausea, and inability to walk despite a National Institutes of Health Stroke Scale (NIHSS) score of 0. Computed tomography angiography (CTA) demonstrated left V3 vertebral artery dissection with pseudoaneurysmal dilation at the craniovertebral junction. Magnetic resonance imaging (MRI) showed a dominant acute left posterior inferior cerebellar artery (PICA)-territory infarction. Cervical imaging demonstrated atlantoaxial deformity, pannus-like peri-odontoid tissue, and a C1 arch abnormality adjacent to the affected V3 segment. Echocardiography and inpatient telemetry did not identify an embolic source. Anatomical colocalization of the C1-C2 abnormality and V3 lesion, together with the ipsilateral PICA infarction, suggests a possible mechanical contribution to the dissection, with subsequent artery-to-artery thromboembolism to the cerebellum. Dynamic compression was not demonstrated; spontaneous dissection, intracranial atherosclerosis, and occult embolism remain alternative or additional mechanisms. The patient received dual antiplatelet therapy followed by aspirin, statin therapy, and rehabilitation. This case emphasizes the limitations of the NIHSS in posterior circulation stroke and the importance of evaluating the craniovertebral junction when a V3 lesion occurs near upper cervical structural disease.

## 1. Introduction

Posterior circulation ischemia may present predominantly with vertigo, nausea, imbalance, truncal ataxia, and inability to walk, while producing few or no points on the National Institutes of Health Stroke Scale (NIHSS). A low NIHSS score therefore does not reliably exclude a disabling cerebellar or brainstem infarction [1].

Vertebral artery dissection can produce stenosis, occlusion, dissecting pseudoaneurysm, local thrombus formation, artery-to-artery embolism, or hemodynamic compromise. Contemporary guidance emphasizes that cervical artery dissection may reflect an interaction among arterial susceptibility, mechanical forces, and local anatomical factors rather than a single uniform mechanism [2]. This interaction is particularly relevant at C1-C2, where the mobile V3 segment courses around the atlas before entering the dura.

Inflammatory or degenerative craniovertebral disease can disturb this relationship through atlantoaxial subluxation, erosive change, peri-odontoid soft tissue, and osseous deformity [3]. Primary reports have described positional vertebral artery occlusion, vertebrobasilar insufficiency, embolic infarction, and vertebral artery dissection in rheumatoid cervical disease [4,5,6,7,8,9,10,11]. Trauma cohorts likewise identify upper cervical fractures and associated subluxation as high-risk configurations for vertebral artery injury [12,13]. Other reports describe dynamically demonstrated V3 compression with dissection or pseudoaneurysmal change caused by atlantoaxial dislocation or a chronic C1 avulsion abnormality [14,15].

We report a patient with a focal left V3 vertebral artery dissection adjacent to chronic C1-C2 structural disease and an ipsilateral PICA-territory infarction. The cervical abnormality may have contributed mechanically to the focal V3 lesion, although motion-dependent compression was not demonstrated. This report follows the CARE case-report guideline, and the authors completed the CARE checklist [16].

## 2. Case Presentation

### 2.1. Reporting Approach and Patient Information

Clinical information, investigations, treatment, and in-hospital outcome were reconstructed from the medical record, and all accompanying images were deidentified. The patient was an older woman with a chart diagnosis of long-standing seronegative inflammatory arthritis and chronic cervical degenerative disease. The available record did not contain sufficient detail regarding the distribution and duration of synovitis, rheumatoid factor or anti-cyclic citrullinated peptide antibody results, erosive disease, longitudinal disease activity, or disease-modifying antirheumatic therapy to independently verify the 2010 American College of Rheumatology/European League Against Rheumatism (ACR/EULAR) classification criteria [17]. Accordingly, we retain the broader term seronegative inflammatory arthritis and do not attribute the craniovertebral abnormality exclusively to rheumatoid disease. Her other relevant conditions included hyperlipidemia, chronic kidney disease, prior peptic-ulcer-related gastrointestinal bleeding, and cognitive impairment attributed to multi-infarct disease.

### 2.2. Clinical Timeline

The sequence of presentation, investigations, treatment, and disposition is summarized in Table 1.

### 2.3. Clinical Presentation and Initial Assessment

She developed abrupt, persistent vertigo, nausea, generalized weakness, poor oral intake, and marked gait instability. The initial examination showed no aphasia, cranial nerve deficit, or focal limb weakness, and the NIHSS score was 0. Nevertheless, she could not mobilize independently because of continuous vertigo, nausea, and imbalance. Intravenous thrombolysis was not administered because the presenting deficits were initially judged non-disabling. Persistence of the acute vestibular syndrome and inability to walk prompted evaluation for a central cause.

### 2.4. Diagnostic Assessment and Differential Diagnosis

**Figure 1:**
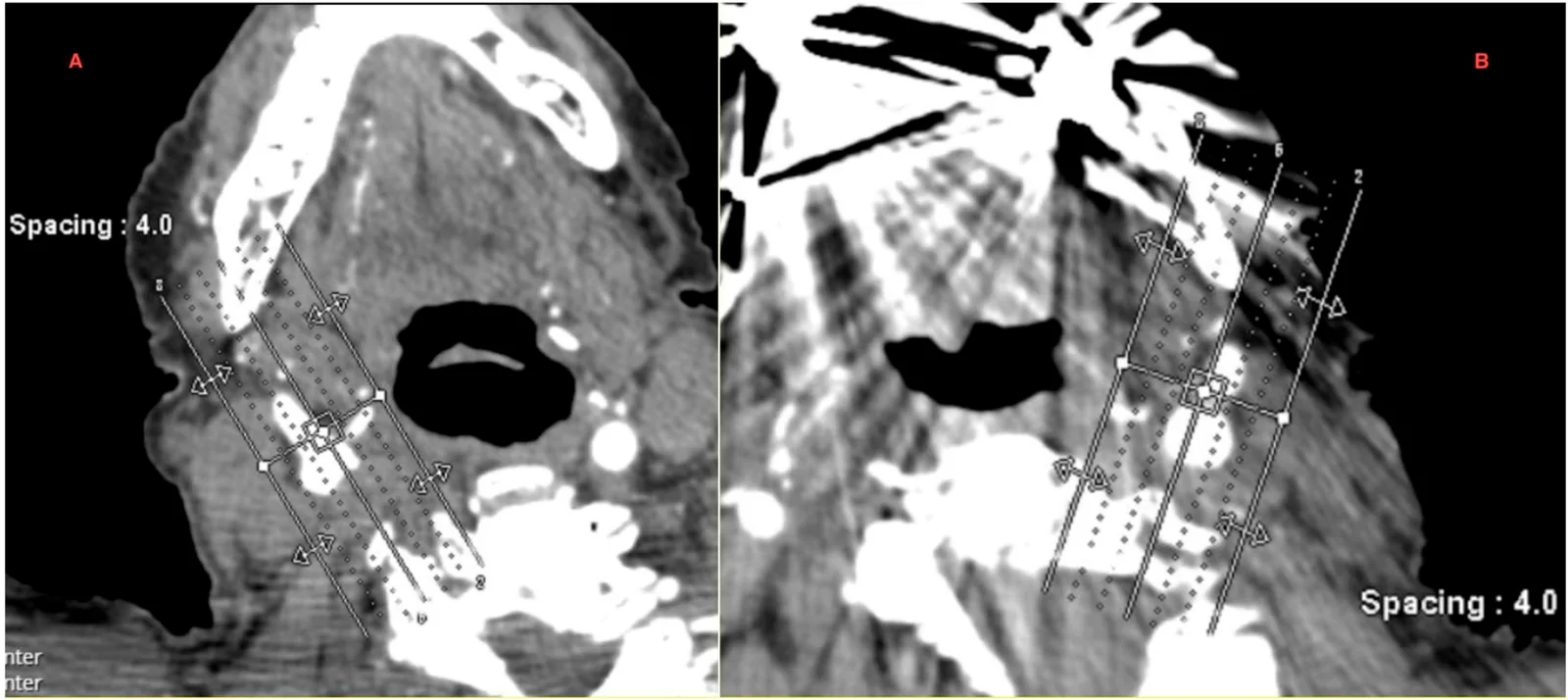
Multiplanar CTA reconstructions of the craniovertebral junction. Panels (**A**,**B**) retain the source-workstation reference lines and plane labels. The CTA study was interpreted clinically as left V3 dissection with pseudoaneurysmal dilation. The displayed workstation overlays partly obscure the vascular contour; these images provide anatomical context and do not independently demonstrate an intimal flap or motion-dependent compression.

Initial noncontrast head computed tomography (CT) showed no acute intracranial hemorrhage, hydrocephalus, or established large infarction. Subsequent computed tomography angiography (CTA) of the head and neck demonstrated focal irregularity of the left V3 vertebral artery with pseudoaneurysmal dilation, interpreted as dissection (Figure 1). CTA also showed several severe intracranial atherosclerotic stenoses and a small incidental anterior communicating artery aneurysm.

**Figure 2:**
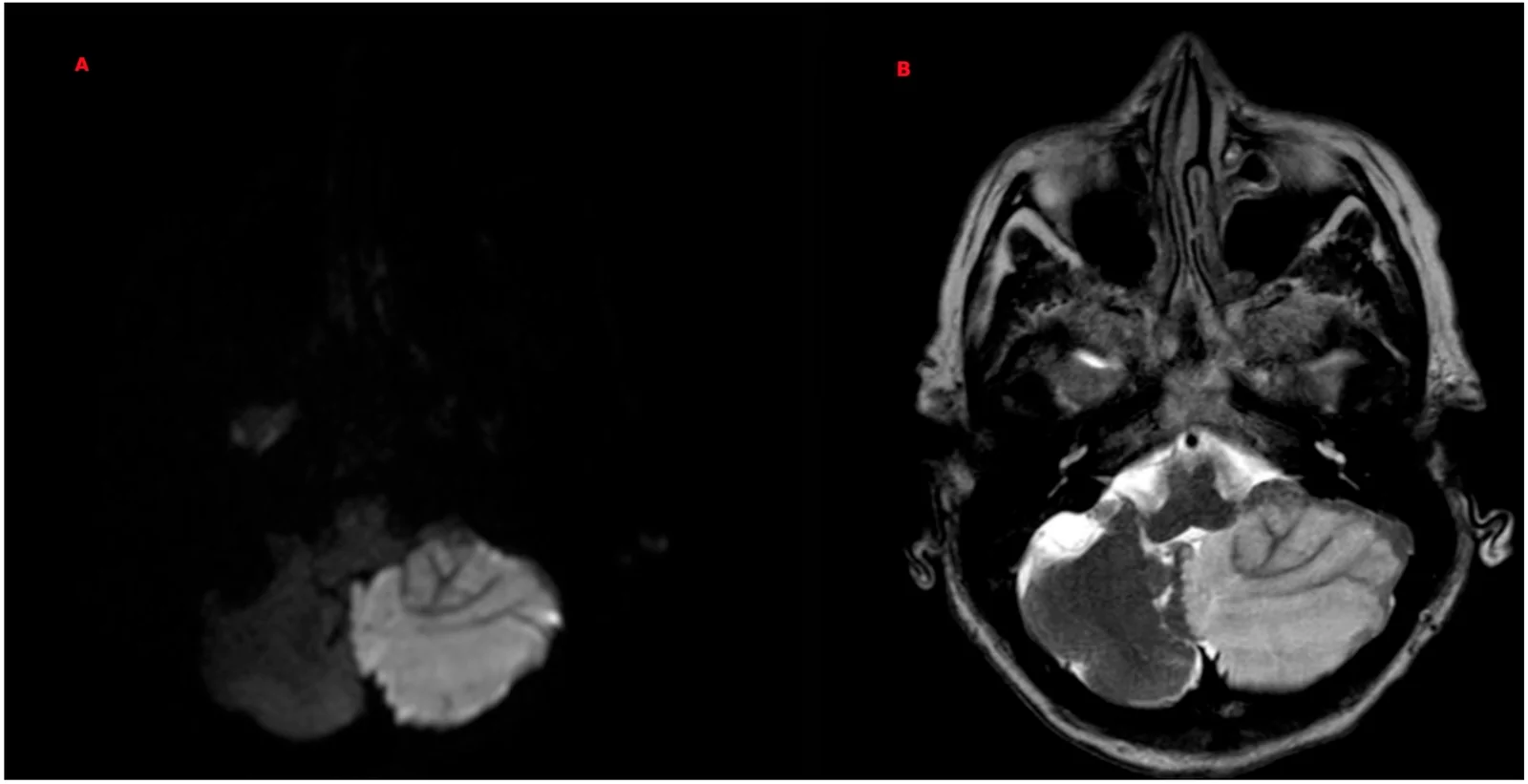
Acute left PICA-territory cerebellar infarction. (**A**) Axial diffusion-weighted MRI shows marked hyperintensity in the left cerebellar hemisphere, consistent with the reported acute infarction. (**B**) Axial T2-weighted MRI shows corresponding hyperintensity and edema. An apparent diffusion coefficient map is not displayed.

Brain magnetic resonance imaging (MRI) demonstrated an acute left PICA-territory cerebellar infarction with edema and mild mass effect on the fourth ventricle, without hydrocephalus (Figure 2). Additional small acute lesions were present in the right cerebellum and left deep white matter/thalamic region. The dominant cerebellar lesion conformed to the left PICA distribution.

**Figure 3:**
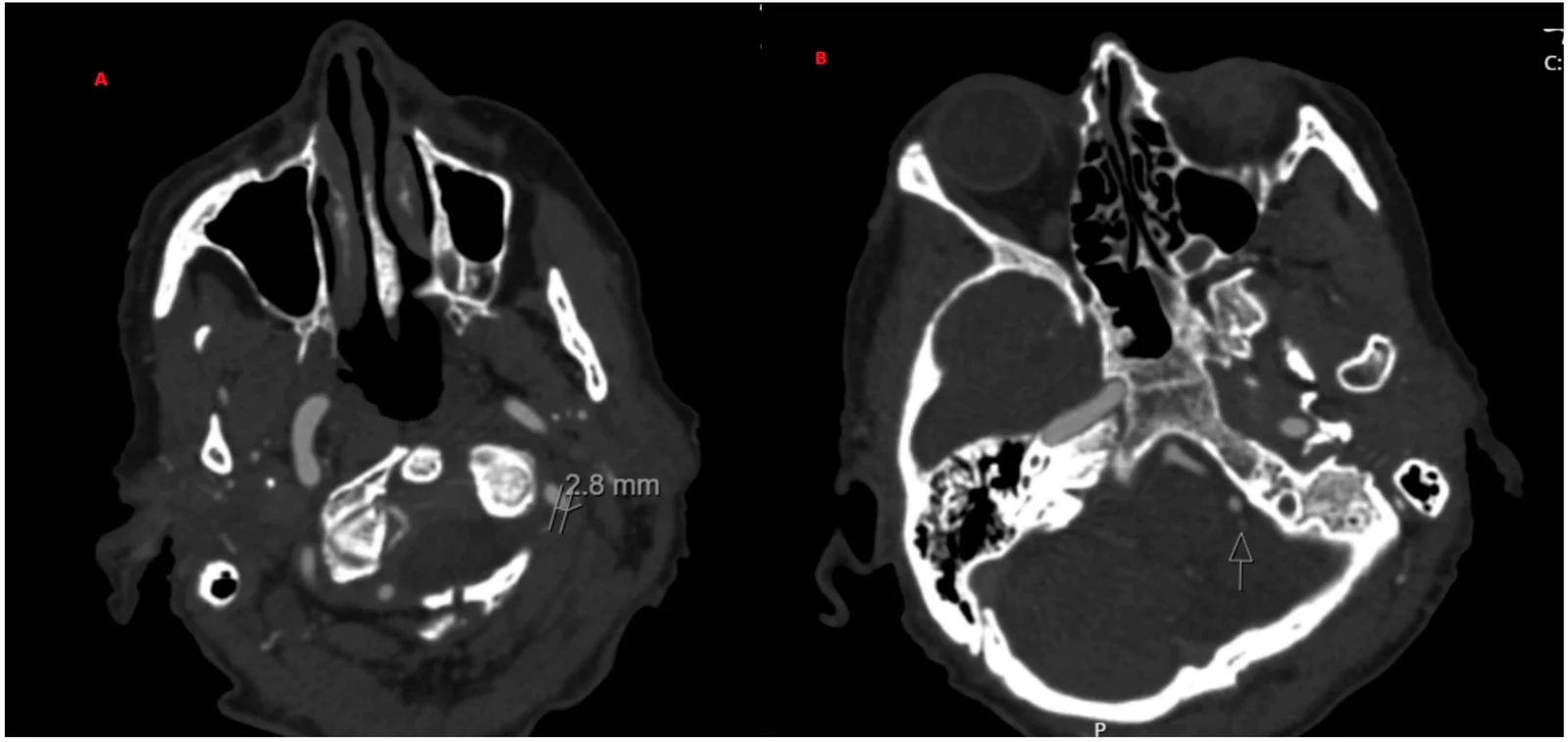
Correlative CT findings. (**A**) Axial CTA source image at C1-C2 shows chronic atlantoaxial deformity, peri-odontoid soft tissue, and a C1 arch abnormality. The 2.8 mm caliper annotation is a source-image measurement in the left V3 region. (**B**) Axial noncontrast head CT shows left cerebellar hypoattenuation (arrow), corresponding to the dominant infarct. P denotes posterior. Peripheral text and annotations are retained from the source images.

Transthoracic echocardiography with bubble study showed a left ventricular ejection fraction of 60–65% without intracardiac thrombus or patent foramen ovale. Glycated hemoglobin was normal, whereas low-density lipoprotein cholesterol was elevated. Hypokalemia was attributed to poor intake and gastrointestinal loss. Cervical imaging demonstrated chronic atlantoaxial deformity, pannus-like peri-odontoid tissue, and a C1 arch abnormality immediately adjacent to the V3 course (Figure 3A). Subsequent head CT demonstrated the corresponding left cerebellar infarction (Figure 3B). This spatial relationship suggests a possible local mechanical contribution, although static imaging cannot demonstrate motion-dependent compression. Results of digital subtraction angiography (DSA), dynamic CTA or magnetic resonance angiography (MRA), and positional duplex testing were not available in the reviewed record. Thus, CTA established the working diagnosis of vertebral artery dissection, whereas the proposed mechanical contribution remained inferential.

Peripheral vestibular disorders, including vestibular neuritis and benign positional vertigo, were considered early but became unlikely after MRI demonstrated an acute cerebellar infarction. The stroke-mechanism differential included spontaneous vertebral artery dissection, artery-to-artery embolism from the dissected segment, intracranial atherosclerotic disease, small-vessel ischemia, and less likely cardioembolism. The negative echocardiogram and inpatient telemetry reduced but did not eliminate an occult cardioembolic source. The focal V3 dissection beside the C1-C2 abnormality and the downstream ipsilateral PICA infarction suggested a possible local mechanical contribution to the dominant lesion; the additional small infarcts and extensive vascular disease supported a potentially multifactorial process.

### 2.5. Treatment

The treating team prescribed dual antiplatelet therapy for 21 days followed by aspirin monotherapy and initiated high-intensity statin therapy. Telemetry did not identify a sustained arrhythmia during hospitalization. Hypokalemia was corrected, and vertigo and nausea were treated symptomatically. Physical, occupational, and speech-language therapy addressed gait, transfers, swallowing safety, and activities of daily living. Neurosurgical review concluded that the chronic craniovertebral disease was not suitable for operative stabilization in the context of age, comorbidity, and nonprogressive structural findings; a cervical collar was not recommended. Posterior fossa decompression was discussed as a contingency should mass effect worsen, but the patient declined this intervention. Goals-of-care discussions resulted in do-not-resuscitate status according to the patient’s preference.

### 2.6. Outcome and Follow-Up

Vertigo partially improved with supportive treatment, but severe gait ataxia and impaired postural control persisted. She required substantial assistance for transfers and short-distance mobility. Serial noncontrast CT scans showed stable edema without hemorrhagic transformation, progressive fourth-ventricle compression, or obstructive hydrocephalus. She was discharged to a post-acute rehabilitation setting for balance retraining, mobility assistance, and fall prevention. Follow-up vascular imaging with CTA or MRA at approximately 3–6 months was recommended to evaluate arterial healing and pseudoaneurysm evolution, with subsequent antithrombotic decisions individualized according to clinical and imaging findings [2].

### 2.7. Patient Perspective

A formal patient-perspective statement was not available for inclusion.

### 2.8. Learning Points

- Persistent acute vestibular symptoms with inability to walk may represent posterior circulation stroke even when the NIHSS score is 0.
- CTA can establish a working diagnosis of vertebral artery dissection, while dynamic vascular imaging or operative evidence can help assess a proposed mechanical contribution.
- A focal V3 lesion immediately adjacent to chronic C1-C2 structural disease, with a downstream ipsilateral PICA infarction, suggests a possible local mechanical contribution even when dynamic compression is not demonstrated.
- When competing vascular mechanisms coexist, the cervical abnormality should be presented as a plausible contributor to the dominant territorial lesion rather than as the exclusive cause of all ischemic findings.
- Seronegative inflammatory arthritis should not be relabeled as rheumatoid arthritis without adequate clinical, serologic, and radiographic support.

## 3. Discussion

### 3.1. Interpretation and Mechanistic Reasoning

**Figure 4:**
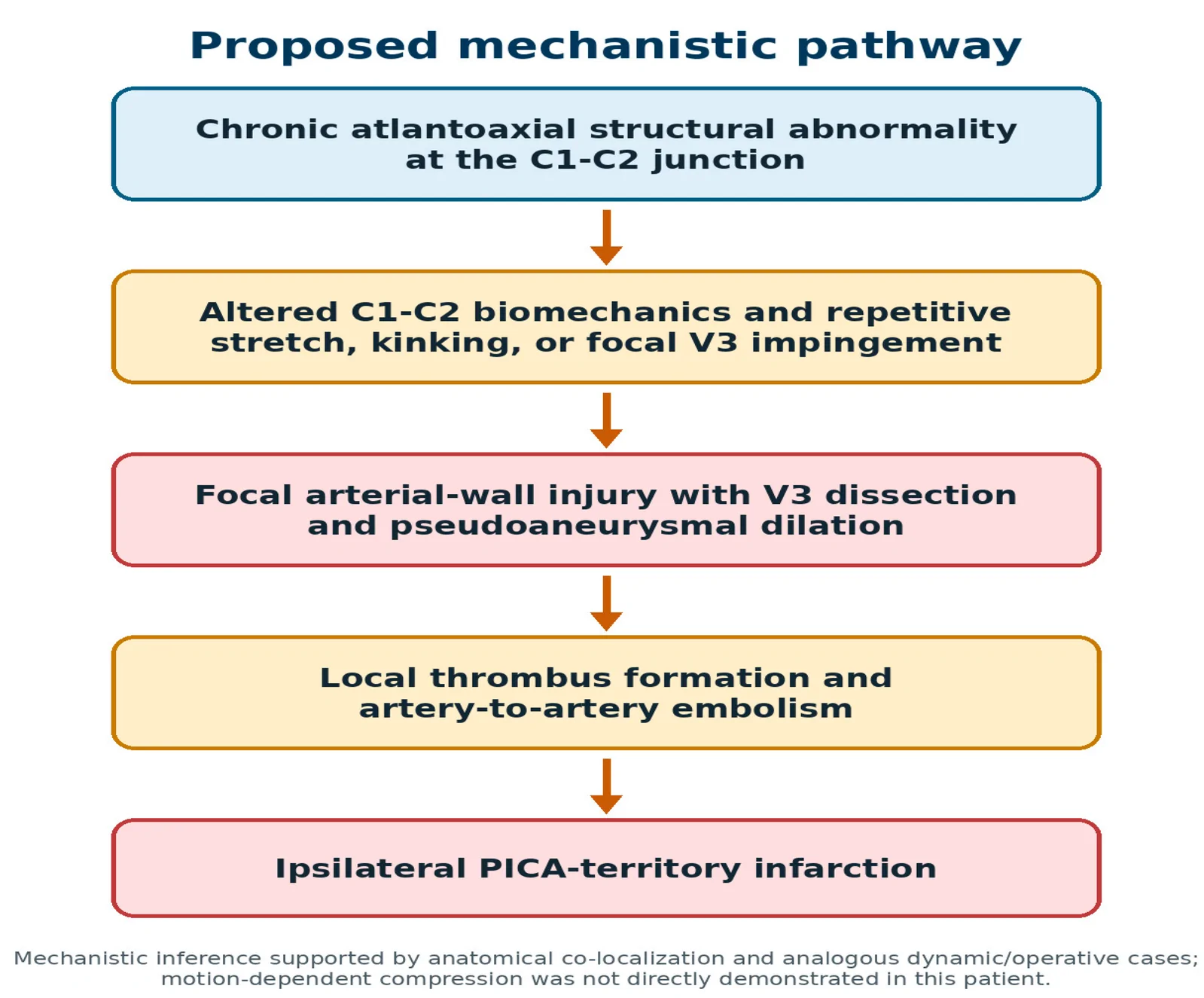
Proposed hypothetical mechanism linking chronic C1-C2 structural disease to posterior circulation infarction. The arrows represent hypothesized causal links. Altered biomechanics, repetitive arterial impingement, local thrombus formation, and artery-to-artery embolism were not directly demonstrated in this patient. Anatomical colocalization and analogous reports support biological plausibility but do not establish causation.

The principal findings were a focal left V3 vertebral artery dissection with pseudoaneurysmal dilation, a dominant ipsilateral PICA-territory infarction, and chronic atlantoaxial structural disease immediately adjacent to the affected arterial segment. The proximity of the osseous and arterial abnormalities and the downstream infarction suggest a possible local mechanical contribution. The hypothesized sequence is altered C1-C2 biomechanics with repetitive V3 stretch, kinking, or focal impingement; arterial-wall injury and dissection; local thrombus formation; and artery-to-artery embolism into the PICA territory (Figure 4).

This interpretation is anatomically coherent but remains an inference. The patient had advanced age, hyperlipidemia, chronic kidney disease, multifocal intracranial stenoses, and prior multi-infarct disease. Her vascular comorbidities provide alternative explanations, including atheroembolism and small-vessel ischemia, for the additional acute lesions. Spontaneous dissection also remains possible. Similarly, negative transthoracic echocardiography and inpatient telemetry did not exclude paroxysmal arrhythmia or another occult embolic source. We therefore consider the chronic C1-C2 abnormality a possible local mechanical substrate for the focal V3 dissection and dominant PICA-territory infarction, within a potentially multifactorial cerebrovascular presentation.

The rheumatologic diagnosis also requires precision. Seronegativity does not exclude rheumatoid arthritis; however, the broader category of seronegative inflammatory arthritis includes several alternative inflammatory arthritides. Without documented joint distribution, serologies, erosions, disease activity, and treatment history, the most accurate description is seronegative inflammatory arthritis with a chronic pannus-like craniovertebral abnormality. Degenerative, inflammatory, post-traumatic, and congenital processes can produce overlapping appearances at C1-C2; the present imaging does not establish a single etiology for the structural disease.

### 3.2. Comparison with Previously Reported Cases

Prior reports establish that abnormal C1-C2 mechanics can compromise the vertebral artery through both hemodynamic and embolic pathways. Rheumatoid atlantoaxial disease has produced position-dependent vertebral artery occlusion, recurrent cerebellar infarction, and focal V3 compression, with clinical improvement or prevention of recurrence after decompression or stabilization [4,5,6,7,8,9,10,11]. Mahajan and Huisa described vertebral artery dissection followed by a left PICA-territory infarction adjacent to severe rheumatoid cervical degeneration, providing a particularly close radiographic analogue [7].

Reports of noninflammatory cervical disease provide further mechanistic context. In a 97-patient atlas-fracture series, vertebral artery injury was independently associated with transverse ligament injury (odds ratio, 8.51), whereas increased atlantodental interval and lateral-mass displacement were associated with ligament disruption [12]. In a cervical-fracture cohort, all five vertebral artery injuries occurred with upper cervical fractures; subluxation and extension into the transverse foramen were highlighted as high-risk configurations [13]. Bando et al. directly demonstrated a V3 dissecting aneurysm with dynamic positional occlusion caused by atlantoaxial rotatory dislocation, followed by freedom from recurrent stroke and reduction of the aneurysm after C1-C2 fusion [14]. Abecassis et al. demonstrated dynamic V3 compression, pseudoaneurysm, and symptomatic stroke associated with a chronic C1 avulsion abnormality; decompression relieved the mechanical compression [15]. These cases show that upper cervical structural disease can produce focal V3 arterial-wall injury and thromboembolic stroke, as well as transient flow limitation. Table 2 compares selected reports with the present case.

### 3.3. Clinical Implications and Limitations

CTA was sufficient to establish a working diagnosis of vertebral artery dissection in this clinical setting, and DSA is not required in every patient when noninvasive imaging is diagnostic and no endovascular procedure is planned [2]. However, rotational or flexion-extension DSA, dynamic CTA or MRA, or positional duplex ultrasonography could have assessed reproducible arterial deformation or flow limitation with neck movement. Operative visualization of arterial contact, improvement after stabilization, or interval change with immobilization would also strengthen the mechanistic inference.

The principal limitations are the single-patient design, incomplete rheumatologic characterization, absence of dynamic vascular imaging and high-resolution vessel-wall MRI, lack of definitive long-term vascular follow-up in the available record, and multiple plausible competing stroke mechanisms. These limitations prevent definitive attribution of the dissection to the C1-C2 abnormality. Anatomical colocalization and prior dynamically confirmed cases support the hypothesis but do not resolve these competing explanations. Serial imaging is also important after a large cerebellar infarction because posterior fossa edema can evolve despite an initially stable examination [18].

## 4. Conclusions

This case describes a focal left V3 vertebral artery dissection with pseudoaneurysmal dilation and an ipsilateral PICA-territory infarction in an older woman with seronegative inflammatory arthritis and chronic atlantoaxial structural abnormality. The proximity of the cervical and vascular lesions suggests a possible mechanical contribution, but dynamic compression was not demonstrated. Spontaneous dissection, intracranial atherosclerosis, small-vessel disease, and occult embolism remain alternative or additional mechanisms. A focal V3 lesion near upper cervical structural disease warrants assessment of the craniovertebral junction. Severe persistent vestibular symptoms and inability to walk should prompt evaluation for posterior circulation stroke even when the NIHSS score is low.

## Figures and Tables

**Table 1:** Clinical timeline reconstructed from the available medical record.

Clinical Phase	Key Events
Presentation	Abrupt persistent vertigo, nausea, generalized weakness, and inability to walk; NIHSS score 0.
Initial diagnostic evaluation	Noncontrast head CT excluded acute hemorrhage. CTA identified focal left V3 dissection with pseudoaneurysmal dilation and chronic C1-C2 structural abnormality.
Inpatient evaluation and course	MRI confirmed a dominant acute left PICA-territory infarction with small additional acute lesions. Echocardiography and telemetry found no clear cardiac embolic source; serial CT showed stable edema without obstructive hydrocephalus.
Treatment and disposition	Dual antiplatelet therapy for 21 days followed by aspirin, high-intensity statin therapy, and multidisciplinary rehabilitation; discharge to post-acute rehabilitation with repeat CTA or MRA recommended at approximately 3–6 months.

**Table 2:** Selected reports of vertebral artery injury or posterior circulation ischemia associated with C1-C2 structural abnormalities.

Report	Patient/Cervical Findings	Vascular and Cerebral Findings	Management/Outcome
Howell and Molyneux, 1988 [4]	Rheumatoid arthritis with atlantoaxial subluxation	Vertebrobasilar insufficiency; angiographic left vertebral artery occlusion	Not reported
Loeb et al., 1993 [5]	A 40-year-old woman with rheumatoid arthritis; vertigo and nausea	Left vertebral artery occlusion demonstrated by angiography and MRI	Not reported
Fujiwara et al., 2012 [6]	Rheumatoid arthritis with reducible atlantoaxial subluxation and recurrent symptoms	Dynamic arteriography showed positional left vertebral artery occlusion; multiple cerebral and cerebellar infarctions	C1-C2 posterior fusion; no recurrent symptoms
Mahajan and Huisa, 2013 [7]	A 59-year-old woman with active rheumatoid arthritis and severe multilevel cervical degeneration	Vertebral artery dissection with left PICA-territory infarction; osteophyte contact proposed	Mechanical mechanism proposed; outcome not reported
Kuroki et al., 2013 [8]	A 78-year-old woman with rheumatoid arthritis and vertical atlantoaxial subluxation	Recurrent embolic cerebellar strokes; bilateral C1-C2 vertebral artery occlusion with distal collateral filling	Association supported by angiographic anatomy; long-term outcome not reported
Takeshima et al., 2015 [9]	A 52-year-old man with rheumatoid arthritis and lateral atlantoaxial subluxation	Multiple vertebrobasilar infarctions associated with lateral atlantoaxial deformity	Surgical stabilization used to address the cervical lesion
Baba et al., 2015 [10]	Two women with rheumatoid destruction of an atlantoaxial joint	Rotation-position 3D-CTA demonstrated vertebral artery occlusion; pontine and cerebellar infarctions	Atlantoaxial/occipitocervical fusion; no recurrent infarctions
Bando et al., 2020 [14]	Atlantoaxial rotatory dislocation with C1 laminar abnormality	V3 dissecting aneurysm and dynamic occlusion during neck rotation; cerebral infarction	C1-C2 fusion; no recurrent stroke and subsequent aneurysm reduction
Curry et al., 2021 [11]	A 72-year-old man with rheumatoid cranial settling and positional syncope/vertigo	Severe left V3 compression between the C1 posterior arch and occiput	Left C1 hemilaminectomy and C1-C4 fusion; positional symptoms resolved
Abecassis et al., 2024 [15]	Chronic C1 avulsion abnormality	Dynamic V3 compression, pseudoaneurysm, and symptomatic stroke	Endoscopic decompression relieved mechanical compression
Present case	Seronegative inflammatory arthritis with chronic atlantoaxial deformity, peri-odontoid tissue, and C1 arch abnormality	Static CTA showed focal left V3 dissection/pseudoaneurysmal dilation; MRI showed dominant ipsilateral PICA-territory infarction	Medical treatment and rehabilitation; plausible mechanical contribution without dynamic confirmation

## Data Availability

All relevant data are included in the article. Additional patient-level data are not publicly available because of patient privacy and confidentiality requirements.
